# Case report: a novel mutation in *ZIC2* in an infant with microcephaly, holoprosencephaly, and arachnoid cyst

**DOI:** 10.1097/MD.0000000000014780

**Published:** 2019-03-08

**Authors:** Jianjun Xiong, Bingwu Xiang, Xiang Chen, Tao Cai

**Affiliations:** a College of Basic Medical Science, Jiujiang University, Jiujiang, Jiangxi; b Physical Medicine and Rehabilitation Center, The Second Affiliated Hospital and Yuying Children's Hospital of Wenzhou Medical University, Zhejiang, China; c Experimental Medicine Section, National Institute of Dental and Craniofacial Research, National Institutes of Health, Bethesda, MD.

**Keywords:** arachnoid cyst, holoprosencephaly, whole-exome sequencing, *ZIC2*

## Abstract

**Rationale:**

Holoprosencephaly (HPE) is a severe congenital brain malformation resulting from failed or incomplete forebrain division in early pregnancy.

**Patient concerns:**

In this study, we reported a 9-month old infant girl with mild microcephaly, semilobor HPE, and arachnoid cyst.

**Diagnoses:**

Potential genetic defects were screened directly using trio-case whole exome sequencing (WES) rather than traditional karyotype, microarray, and Sanger sequencing of select genes.

**Outcomes:**

A previous unpublished de novo missense mutation (c.1069C >G, p.H357D) in the 3rd zinc finger domain (ZFD3) of the *ZIC2* gene was identified in the affected individual, but not in the parents. Sanger sequencing using specific primers verified the mutation. Extensive bioinformatics analysis confirmed the pathogenicity of this extremely rare mutation. Phenotype-genotype analysis revealed significant correlation between the 3rd zinc-finger domain with semilobor HPE.

**Lessons:**

These findings expanded the spectrum of the *ZIC2* gene mutations and associated clinical manifestations, which is the first identification of a mutated *ZIC2* gene in a Han infant girl with mild microcephaly, semilobor HPE, and arachnoid cyst.

## 1 Introduction

Holoprosencephaly (HPE; MIM: 236100) is the common structural malformation of human forebrain and due to failed cleavage of the developing brain during early pregnancy.^[1,2]^ According to the severity, classical HEP is characterized to 3 subtypes (alobar form, semilobar form, and lobar form). The alobar form is the most serious subtype, in which the brain is not divided into hemispheres at all. By contrast, the semilobar form is characterized by an incomplete forebrain division, which is intermediate in severity. The least severe form is lobar HPE, characterized by the presence of the inter-hemispheric fissure along almost the entire midline hemispheres.^[3]^ In addition, there is a nonclassical HPE named middle interhemispheric variant (MIHV), which is characterized a failure division of the posterior frontal and parietal lobes.^[4]^ Besides organic brain damaging, HPE pathogenesis is usually accompanied by several specific clinical features, including neurological impairment, seizures, diabetes insipidus, characteristic dysmorphic facies, and so on.^[5]^ In common cases, affected individuals with significant craniofacial anomalies correlate with severe brain malformation, which is named as “the face predicts the brain”.^[6]^

The survival infant rate with HPE is 1 per 10,000 to 16,000 by birth.^[7]^ Both genetic and environmental factors are contributed to its occurrence. Mutations in a dozen of genes have been reported to cause this disease, and 4 of them are identified as the “major” causal genes due to high-frequency mutation, including SHH (Sonic Hedgehog, MIM: 600725), *ZIC2* (Zic Family Member 2, MIM: 603073), TGIF (TGFB Induced Factor Homeobox 1, MIM: 602630), and SIX3 (SIX Homeobox 3, MIM: 603714).^[8–11]^

*ZIC2* encodes a member of zinc finger proteins, which is especially expressed in cerebellum at high levels.^[12]^ As a transcriptional repressor, ZIC2 plays a crucial role in neurological development.^[13]^ In early embryo stage, mouse *Zic2* functions in axial midline establishment and then affects the development of the dorsal telencephalon.^[14]^ In human, *ZIC2* is the second frequently mutated gene responsible for >3% of HPE; about 90% of *ZIC2* mutations have been found to lead to structural brain impairments.^[5]^ To date, over 100 different variants in *ZIC2* gene have been confirmed to induce HPE human gene mutation database (HGMD). However, no causal mutations in *ZIC2* have been reported, to best of our knowledge, in affected individuals with HPE in Chinese Han population.

Trio-based whole exome sequence (WES) has been used as an efficient genetic tool to identify de novo causative mutations for clinical diagnosis, as shown in our recent reports on various brain development-related conditions.^[15,16]^ In the present study, we report a previously undescribed missense mutation in the *ZIC2* gene that is identified in an infant girl with HPE and arachnoid cyst.

## 2 Methods

### 2.1 Ethical approval and consent

The affected 9-month-old infant girl was the first child in the family. Her medical records were provided by Second Affiliated Hospital and Yuying Children's Hospital of Wenzhou Medical University. Informed written consent was obtained from the parents for publication of this case report and accompanying images; associated study was authorized by the ethical committees of Wenzhou Medical University.

### 2.2 Trio-WES and Sanger sequencing

Intact DNA was extracted from peripheral blood cells. Whole-exome was captured by SureSelect Human All Exon Kit (Agilent), followed high-throughput sequencing by HiSeq2000 sequencer (Illumina Inc.) The reads were aligned for SNP calling and analysis for candidate genes. The variants recorded in the dbSNP, HapMap, 1000 Genomes, exome aggregation consortium (ExAc), and in-house Chinese Exome Database with minor allele frequency (MAF) >0.001 were deleted. Candidate variant was verified by PCR and ABI 3730 DNA sequencer with mutation-specific primers.

### 2.3 Bioinformatic analysis

Detrimental missense single nucleotide variants (SNVs) were predicted by

(1) Scale-invariant feature transform (SIFT) (sift.bii.astar.edu.sg): a SNP with SIFT score <0.05 predicts a harmful effect on the protein function;
(2) Polyphen-2 (genetics.bwh.harvard.edu/pph2): a SNP with score between 0.85 and 1.0 predicts disease-causing;
(3) Mutation Taster (www.mutationtaster.org): the score close to 1 indicates to be morbific.

MEGA software was performed to analysis multiple-sequence alignment and conservation. 3D structure of ZIC2 protein was depicted by SWISS-MODEL (http://swissmodel.expasy.org/).

## 3 Case report

### 3.1 Clinical features

**Figure 1:**
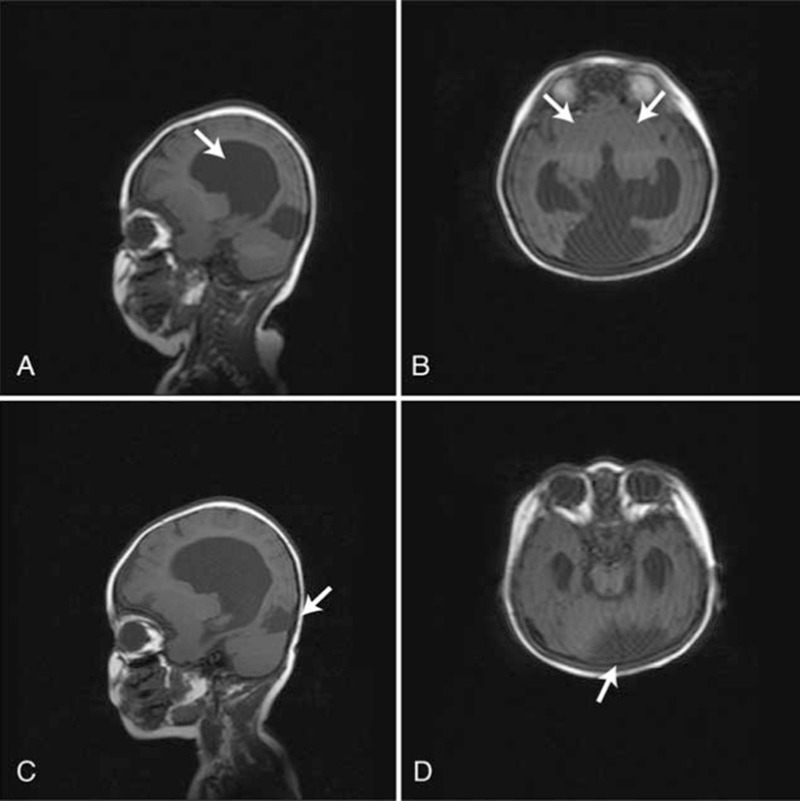
MRI examination of the affected individual with brain malformation. (A) Sagittal section of T1W1 shows deficient sickle, septum and corpus callosum in brain; (B) Axial T1-weighted image shows deficient sickle, septum and corpus callosum in brain; (C) Sagittal section of T1W1shows a cystic abnormal signal shadow; (D) Axial T1-weighted image shows a cystic abnormal signal shadow (arrow). MRI = magnetic resonance imaging.

A 9-month-old infant was diagnosed as developmental delay (DD). She was born after 41 weeks of pregnancy as the first child of healthy and nonconsanguineous parents. One week before birth, the fetus was diagnosed as “hydrocephalus” by ultra-sound. Her bodyweight was 3400 g at birth. She began to hold her head up at 7 months old in prone position, but not stable. At the age of 8 months, she was unable to turn over and to sit. At 9 months old, her head circumference was 41 cm (average size in 9-month normal females: 44.5 cm, ranging 42.1∼46.9 cm). Her knees were hyperreflexia and asymmetric tonic neck reflex was positive. Cranial MRI inspection showed cerebella atrophy and enlargement of bilateral ventricles. The anterior part of the brain was deficient in sickle, septum and corpus callosum. Also, bilateral frontal lobes were fused together, and bilateral ventricles were interlinked to form a single ventricle in the shape of riding boots (Fig. 1A and B). Notably, an abnormal cystic signal shadow (52 × 36 mm in size) was observed in the occipital region (Fig. 1C and D), showing clear boundary and even internal signal near the cerebella curtain. According to the MRI analysis, the affected infant was diagnosed having semilobar HPE and arachnoid cysts. The parents were phenotypically normal.

### 3.2 Genetic analysis

**Figure 2:**
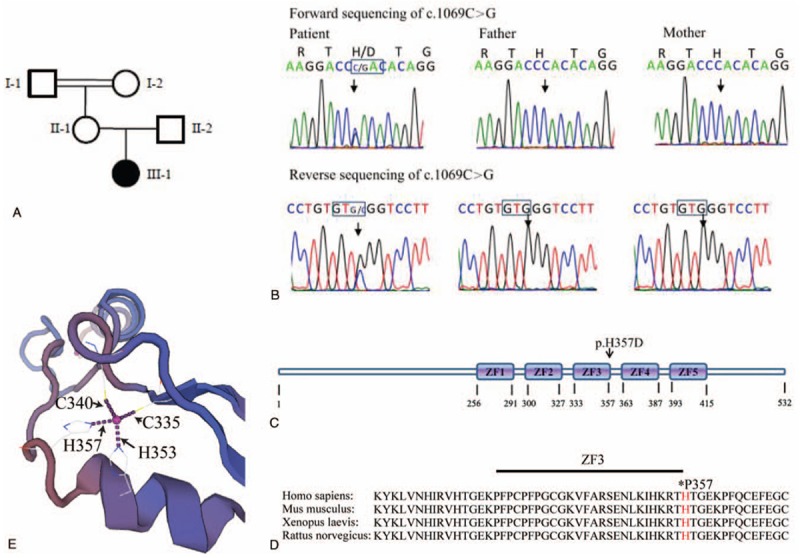
A de novo missense mutation of *ZIC2* is identified. (A) The pedigree of the family; (B) The variant c.1069C >G is confirmed by bidirectional Sanger sequencing. (C) The resulted missense mutation p.H357D is located in the 3rd ZFD of the ZIC2 protein. (D) The amino acid residue 357 Histidine of ZIC2is evolutionarily conserved crossingall vertebrates we examined. (E) The interactionsinvolving amino acid C335, C340, H353, and H357 are predicted by SWISS-MODEL program. *ZIC2* = Zic Family Member 2.

Trio-WES analysis of the family, including the patient and her parents, identified a previously unpublished de novo heterozygous variant (c.1069C >G, p.H357D) in the first exon of the *ZIC2* gene (GenBank, NM_007129.4). This rare variant was confirmed by Sanger sequencing in the proband but not present in the parents (Fig. 2A and B). This substitution of Histidine with Aspartic acid at residue 357 is within the 3rd zinc finger domain (ZFD3) of the *ZIC2*- encoded protein. Multiple-sequence alignment showed that the ZFD3 is evolutionarily conserved in all vertebrates we examined (Fig. 2C and D), suggesting its functional importance.

Furthermore, this variant (c.1069C >G, p.H357D) was predicted to be pathogenic by several bioinformatic tools, including SIFT, polyphen-2, and Mutation Taster. According to SWISS-MODEL prediction, substitution of the Histidine by Aspartic acid could disrupt the interactions involving the amino acid residual C335, C340, H353, and H357, which may affect the structural confirmation and charge of the protein (Fig. 2E).

### 3.3 Genotype–phenotype correlation

Current HGMD curated 113 previously published genomic alterations of *ZIC2*-containing region, including 24 missense mutations (21.2% of total variants, Table 1), 14 nonsense mutations, 4 splicing mutations, 7 mutations in regulatory region, 33 small deletions, 13 small insertions, 5 small indels mutations, and 7 gross deletions mutations. Interestingly, most of reported missense mutations (19/24) are located within zinc-finger domain (ZFD) 1, 2, and 5. Only 2 missense mutationsare found in the ZFD3, including the present novel allele and apreviously reported (c.1004G >T; p.C335F, Table 1). Genotype–phenotype correlation analysis on all 25 cases with missense mutations, 17 of them was categorized into 3 different subtypes of brain images: 6 in the alobar form, 7 in the semilobar form and 4 in the lobar form. We noticed that most of the alobar forms result from mutations within the *N*-terminal region of *ZIC2*, while a majority of the semilobar form is associated with the *C*-terminal counterpart.

**Table 1:**
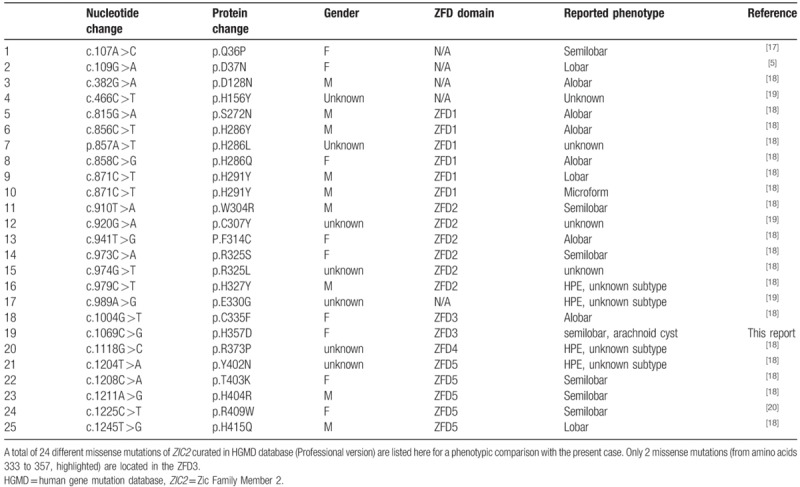
Genotype-phenotype analysis of missense mutations in ZIC2.

## 4 Discussion

The phenotypic spectrum of HPE has been widely presented due to heterogenous genetic defects of more than a dozen identified causal genes. WES is believed to be an effective approach for identification of genomic defects in affected individuals with this disease.^[17]^ Using the trio-WES method, we quickly identified a previously undescribed de novo mutation of *ZIC2* in an infant with semilobar HPE, which may represent the first case with *ZIC2* mutation in Han population.

As a member of ZIC family of zinc finger proteins, ZIC2 plays a crucial role in brain development in embryonic stages.^[18]^ Since the first mutation of the *ZIC2* gene was reported to cause human HPE in 1998, increasing studies show that it is one of the most commonly HPE-associated genes. Although most *ZIC2* mutations are found to be loss-of-function alleles,^[5]^ 13 different missense mutations in the ZFDs have been confirmed to be sufficient to cause HPE (HGMD), suggesting an important role of ZFDs in ZIC2 function. Protein structural analysis reveals 5 evolutionarily conserved ZFD domains, involving protein-protein interaction, DNA binding for transcriptional activation, and nuclear localization.^[19]^ Considering only a single missense mutation was previously identified in ZFD3, one may argue that any given deleterious missense mutations in this highly conversed domain may result in more severe phenotypes during earlier development. In fact, the substitute of Histidine by Aspartic acid may affect the confirmation and charge of the protein, as predicted to be deleterious by multiple commonly used algorithms. It is worth noting that the phenotype of the previous case with a missense mutation (c.1004G >T, Table 1) within the ZFD3 is different from the current case, suggesting the complexity of the ZFD3 function.

Although patients with significant craniofacial anomalies correlate with severe brain malformation in HPE cases,^[6]^ the present case showed a mild microcephaly, but no facial abnormalities. In fact, many affected individuals with mutations of *ZIC2* were previously reported to have minor facial manifestations, such as bitemporal narrowing, upslanted palpebral fissures, a flat nasal bridge, and nasal cleft.^[20]^ More severe facial abnormalities are often observed in cases with mutations in other HPE-causal genes, such as *SHH*, *SIX3,* and *TGIF*.^[21]^ On the other hand, de novo mutations in *ZIC2* are more frequently identified in clinical cohorts, compared to the inherited mutations detected in *SHH* and *SIX3*.^[22]^ The lower inherited mutation rate in *ZIC2* may attributed to a higher mortality rate in affected individuals.

Arachnoid cyst, to best of our knowledge, has not been reported in patients with *ZIC2* mutations. After trauma history is excluded in given cases, arachnoid cysts could result from autosomal inheritance or other factors. As shown in HGMD, mutations in *FOXC2* and *HOXD4* can lead to spinal extradural arachnoid cysts.^[23,24]^ Interestingly, *Zic2a2b* knockdown in zebrafish was found to lead to reduced expression of the *Hoxd4a* gene, which has been proved to be associated with arachnoid.^[25]^ To verify the association between *ZIC2* mutations and arachnoid cysts, however, more genetic evidence is needed from patients with similar manifestations, like previously reported siblings with arachnoid cysts, microcephaly and developmental delay symptoms.^[26]^ Additional factors like environmental effects were also proposed to be the triggers for HPE.^[27]^

In summary, our trio-WES and genetic analysis demonstrate that the c.1069C >G (p.H357D) heterozygous mutation in *ZIC2* is a novel genetic allele of HPE. To our knowledge, this is the first identification of *ZIC2* mutation-associated HPE in Han population. These findings expand the phenotypic spectrum of *ZIC2*-associated disorders.

## Acknowledgments

The authors are thankful for the participation of the family in this study.

## Author contributions

**Data curation:** Jianjun Xiong, Bingwu Xiang, Xiang Chen.

**Formal analysis:** Jianjun Xiong, Tao Cai.

**Funding acquisition:** Jianjun Xiong, Xiang Chen, Bingwu Xiang.

**Investigation:** Jianjun Xiong, Xiang Chen.

**Project administration:** Jianjun Xiong, Xiang Chen, Tao Cai.

**Resources:** Jianjun Xiong, Bingwu Xiang, Xiang Chen.

**Supervision:** Tao Cai.

**Writing – original draft:** Jianjun Xiong.

**Writing – review & editing:** Tao Cai.
